# Investigating Structural Brain Changes of Dehydration Using Voxel-Based Morphometry

**DOI:** 10.1371/journal.pone.0044195

**Published:** 2012-08-29

**Authors:** Daniel-Paolo Streitbürger, Harald E. Möller, Marc Tittgemeyer, Margret Hund-Georgiadis, Matthias L. Schroeter, Karsten Mueller

**Affiliations:** 1 Max Planck Institute for Human Cognitive and Brain Sciences, Leipzig, Germany; 2 Max Planck Institute for Neurological Research, Cologne, Germany; 3 Clinic for Cognitive Neurology, University of Leipzig, Leipzig, Germany; University of Regensburg, Germany

## Abstract

Dehydration can affect the volume of brain structures, which might imply a confound in volumetric and morphometric studies of normal or diseased brain. Six young, healthy volunteers were repeatedly investigated using three-dimensional *T*_1_-weighted magnetic resonance imaging during states of normal hydration, hyperhydration, and dehydration to assess volume changes in gray matter (GM), white matter (WM), and cerebrospinal fluid (CSF). The datasets were analyzed using voxel-based morphometry (VBM), a widely used voxel-wise statistical analysis tool, FreeSurfer, a fully automated volumetric segmentation measure, and SIENAr a longitudinal brain-change detection algorithm. A significant decrease of GM and WM volume associated with dehydration was found in various brain regions, most prominently, in temporal and sub-gyral parietal areas, in the left inferior orbito-frontal region, and in the extra-nuclear region. Moreover, we found consistent increases in CSF, that is, an expansion of the ventricular system affecting both lateral ventricles, the third, and the fourth ventricle. Similar degrees of shrinkage in WM volume and increase of the ventricular system have been reported in studies of mild cognitive impairment or Alzheime 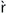
s disease during disease progression. Based on these findings, a potential confound in GM and WM or ventricular volume studies due to the subjects’ hydration state cannot be excluded and should be appropriately addressed in morphometric studies of the brain.

## Introduction

A number of recent studies employing structural magnetic resonance imaging (MRI) have aimed to investigate gray matter (GM), white matter (WM), and the cerebrospinal fluid (CSF) system and its explanatory power for neurodegenerative disorders [1]–[3]. Regarding Alzheimer’s disease (AD), decreased GM and WM volumes were consistently found [4], [5], which are assumed to be related to the loss of neurons and synapses. This, in turn, could also be a plausible explanation for an accompanied increase of CSF as several studies showed an enlarged size of the ventricles due to brain atrophy compared to healthy controls [2], [6], [7]. Annual expansion of the ventricles in healthy elderly and AD patients is around 1.5–3.0% and 5–16%, respectively [8].

Such changes in brain tissue and fluids led to the idea of disease progression measures based on different approaches and tissue types [3]. Nestor et al. [2] proposed ventricular enlargement as a valid and sensitive short-term marker of disease progression in subjects with AD and mild cognitive impairment (MCI) for multi-center studies.

Besides investigations of CSF in dementia, several other studies showed CSF volume changes using VBM in non-neurodegenerative diseases. For example, Bendel et al. [9] demonstrated a correlation of neuropsychological outcome after subarachnoid hemorrhages with an enlargement of CSF volume. They claimed that ventricular enlargement combined with GM loss may indicate general brain atrophy rather than hydrocephalus. Enlarged CSF and decreased GM volumes have also been found in women with schizotypal personality disorder [10]. Furthermore, Hagemann et al. [11] presented a strong correlation of progesterone and CSF volume change during the menstrual cycle. Consequently, they recommended considering such short-term hormone-dependent structural brain changes in VBM studies.

However, CSF can also be influenced intentionally by massive fluid intake [12] and nonintentionally by external factors, such as high ambient temperature or acute physical stress due to illness, infections, or fever [13]. Recent studies have thus pointed to a potential confound in morphometric MRI studies due to a continuous and severe lack of hydration [12], [14]–[16]. For example, dehydration might produce an additive effect in AD studies because increases of CSF volume, in particular in the ventricles, might be a result of long-term dehydration instead of degeneration of brain tissue.

Considering intentional influence of fluid balance by water intake, Kempton et al. [16] observed significant ventricular changes in acute dehydration with structural MRI. In particular, they found enlarged lateral ventricles but no changes in the fourth ventricle, although the whole ventricular system is predicted to be affected by variation in hydration status. Similarly, Duning et al. [12] showed structural effects due to rehydration after dehydration, using a water intake of 1.5 l. This effect might be helpful to “normalize” subjects’ fluid balance by changing from an often poorly controlled (and, hence, more variable) ‘normal hydration’ state to a more consistent ‘hyperhydration’ state.

Dickson et al. [14], Duning et al. [12], and Kempton et al. [16] used the fully automatic method SIENA [17] as well as the manual segmentation tools MEASURE [18] and Analyze in their studies. SIENA is specifically designed to detect small morphological brain changes in longitudinal MRI studies. It gains its sensitivity by edge motion detection and maps changes onto surrounding edges. This approach is not accurate on spatial information and does not specify locations of detected volume changes. Another often-used method to detect subtle structural changes in cross-sectional studies is voxel-based morphometry (VBM) [19]. It permits a longitudinal preprocessing approach that is not limited to edge detections and allows to assess structural changes for the entire brain.

In our present study, we investigate if dehydration effects can be measured in a longitudinal VBM study. Based on previous studies, we hypothesized that dehydration leads to an enlargement of the ventricular system. As currently detailed investigations of a potential influence on GM and WM are lacking, we additionally hypothesize a decrease of GM and/or WM volumes due to dehydration.

## Methods

### 2.1 Subjects and Imaging Procedures

Six healthy young adults (3 female; mean 24.7±3.0 y, all right handed) participated in a long-term hydration experiment. All participants gave written consent after being informed about the possible risks and discomforts of the experimental procedure. Subjects also completed a health history questionnaire to assess their suitability for undergoing MRI scanning. Imaging was performed on a 3-T MAGNETOM Trio scanner (Siemens Medical Solutions, Erlangen, Germany) with a birdcage transmit/receive head coil. *T*_1_-weighted images were acquired with a three-dimensional MP-RAGE sequence using the following parameters: inversion time 650 ms; repetition time, *TR*  = 1.3 s; *TR* of the gradient-echo kernel 10 ms; echo time 3.93 ms, flip angle 10°, bandwidth 130 Hz/pixel, acquisition matrix 256×240, field of view 256×240 mm^2^, slab thickness 192 mm (sagittal orientation), 128 partitions, 95% slice resolution, 2 averages. After zero filling, reconstructed images were obtained with a nominal voxel size of 1×1×1 mm^3^.

### 2.2 Study Protocol

Subjects had to follow a strict hydrating/thirsting protocol, in particular, they were instructed not to participate in strenuous activity and to avoid alcohol consumption during the three days of the study. On subjects’ arrival (between 8∶00 and 9∶00 in the morning), image data were acquired in a ‘normal hydration’ state. The time of this scan is subsequently referred to as *t*  = 0. We note that there were no restrictions or specific requirements regarding fluid or food intake prior to this scan, which hence reflects the natural variability of water balance in subjects recruited for typical MRI studies. Afterwards, all subjects were instructed to drink at least 3–4 l of water and were scanned again at *t* ≈ 10 h (subsequently referred to as ‘hyperhydration state’). A summary of individual water intake between the first two scans is given in Table 1. For comparison, the mean daily fluid intake in healthy male adults is approximately 2.1 l [20]. For the next two days (i.e. days 2 and 3), subjects were allowed to drink 150 ml of water per day and had to avoid meals with a high fluid content. Gullans and Verbalis [21] described a steady decline of the dehydration effect with time in a rat study. Therefore we decided to acquire three scans on day 3 to allow additional investigation of dynamic changes during dehydration. These three scans (subsequently referred to as ‘dehydration scans’) were acquired at times *t* ≈ 48 h, 53 h, and 58 h after the ‘normal hydration’ scan (i.e. 38 h, 43 h, and 48 h after the ‘hyperhydration’ scan. Throughout the entire study, body weight, daily urine flow, and meal consumption (restricted to bread, rice, and potatoes on days 2 and 3) were monitored to ensure participants had adhered to the protocol. A summary of individual variations in urinary excretion and body weight is given in Table 2. On average, subjects lost approximately 2.3% of their body weight between the first and last scan. On day 3, they had an average urinary excretion of 908 ml as compared to an average value of approximately 1.3 l in healthy subjects under normal conditions [20].

**Table 1:** Subjects’ water intake, *V_w_*, between the scans performed at normal hydration (*t* = 0) and the hyperhydration (*t* = 10 h) on day 1.

Subject	*V_w_* [ml]
1	3600
2	3950
3	3300
4	4100
5	3750
6	4200

**Table 2:** Subjects’ cumulative urinary excretion between successive time points and body weight during the complete study.

Subject	Day 1			Day 2			Day 3			
	*t* = 0	*t* = 5 h	*t* = 10 h	*t* = 24 h	*t* = 29 h	*t* = 34 h	*t* = 48 h	*t* = 53 h	*t* = 58 h	
Urine excretion, Δ*V_u_* [ml]										
1		1500	3500	800	0	100	300	0	150	
2		1300	3000	0	230	400	700	190	370	
3		1000	3250	700	130	300	330	180	280	
4		1100	3100	0	0	300	900	0	250	
5		1500	3000	550	100	250	550	100	150	
6		1500	3500	0	0	300	850	150	0	
Body weight, *M* [kg]										
1	56.7	57.0	56.7	55.8	55.4	55.6	56.2	54.7	54.9	
2	63.5	64.3	64.8	63.4	63.4	63.2	63.1	63.0	62.8	
3	57.0	58.0	57.3	56.5	56.6	56.4	56.0	55.8	55.8	
4	59.1	60.1	61.1	59.1	59.4	59.3	58.6	58.7	58.4	
5	74.2	75.0	74.9	74.4	74.0	74.1	73.5	73.5	73.3	
6	80.2	81.0	80.6	78.1	78.0	77.4	76.3	76.2	75.7	

The time points of the five MRI scans are indicated in bold.

### 2.3 Voxel-Based Morphometry

Images were processed using the longitudinal processing pipeline as offered in the VBM8 toolbox (Gaser, C., http://dbm.neuro.uni-jena.de/vbm/, last accessed 07.09.2011). Segmented GM, WM, and CSF images were smoothed with 8 mm^3^ full width at half maximum and fed into a flexible factorial design with two factors (subject and hydration state). Assignments to the different levels of hydration state were: ‘0’ for normal hydration at *t* = 0, ‘–3’ for hyperhydration at *t* = 10 h, and ‘+1’ for dehydration at *t* = 48 h, 53 h, and 58 h. Additionally, statistical computations with assignments ‘0′ for normal hydration, ‘–6’ for hyperhydration, and ‘+3, +2, and +1’ for the scans during dehydration at *t* = 48 h, 53 h, and 58 h, respectively, were calculated in order to investigate dynamic changes during dehydration.

No potentially confounding variables were included. Smoothed GM, WM, and CSF images were thresholded excluding voxels containing a probability density below 10%. Finally, non-stationary cluster extent corrections [22], [23] were applied to the VBM results.

### 2.4 SIENAr

The longitudinal voxel-wise statistical edge motion detection approach SIENAr implemented, published and provided by FSL, was applied to our data. Default parameters as described on the software website ([17], http://www.fmrib.ox.ac.uk, last accessed 08.01.2012) were chosen and comparisons of hyperhydration and the first dehydration state were statistically assessed using one-sample *t*-tests with the randomise software of FSL [24].

### 2.5 FreeSurfer

Both lateral ventricles as well as the third and fourth ventricle were segmented using the FreeSurfer image analysis suite (version. 4.5) [25] with default parameters on a Debian 5.0 system. Summarized segmentation results were fed into a repeated measurements ANOVA using SPSS version 19.0 (IBM SPSS Inc., Chicago, IL, USA). Based on prior knowledge, one-tailed paired *t*-tests were computed comparing normal hydration, hyperhydration, and each of the dehydration datasets. Furthermore, cortical thickness data, a result of the FreeSurfer image processing pipeline, were smoothed with a 20-mm Gaussian kernel and statistically assessed in a similar fashion as the VBM-processed data. In particular, a mixed-effects model using the SurfStat software [26] was modeled assuming thinning in dehydration and thickening in hyperhydration.

## Results

**Figure 1:**
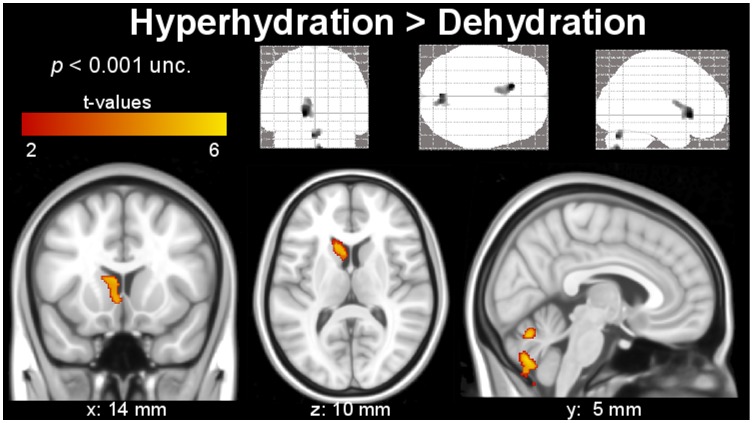
Segmented clusters of the gray matter with significant extension during hyperhydration compared to dehydration in caudate nucleus and cerebellar regions (indicated by the color code) obtained with VBM. The upper row shows the results as grayscale Maximum Intensity Projection onto the standard SPM glass brain in coronal, axial and sagittal view.

**Figure 2:**
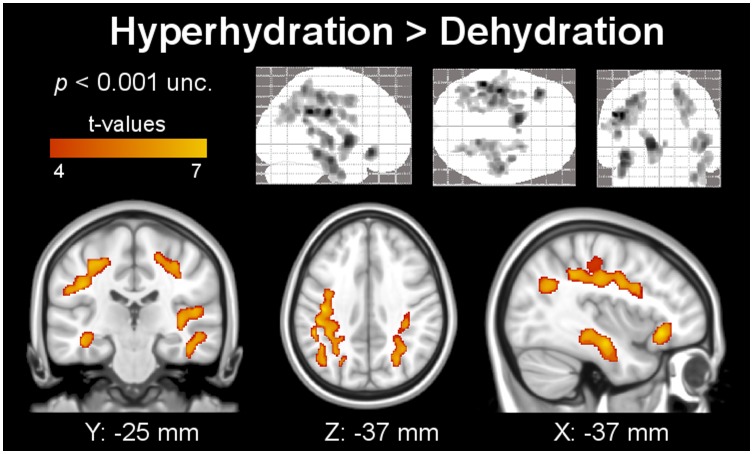
Segmented clusters of the white matter with significant extension during hyperhydration compared to dehydration in parietal and tempo-parietal regions (indicated by the color code) obtained using VBM. The upper row shows the results as grayscale Maximum Intensity Projection onto the standard SPM glass brain in coronal, axial and sagittal view.

**Figure 3:**
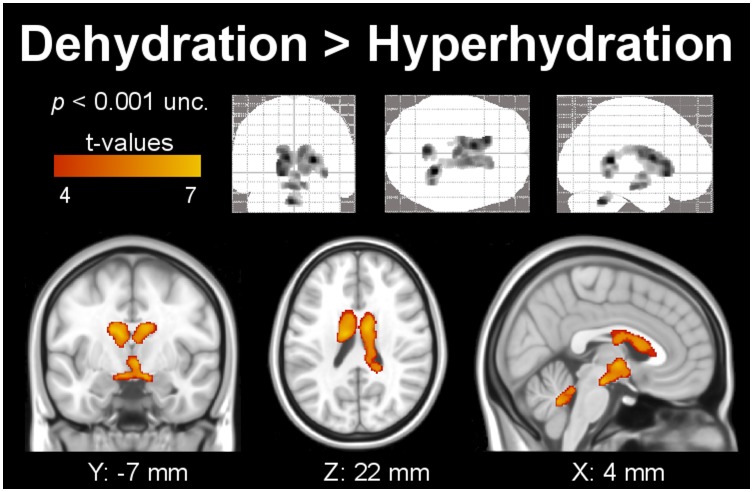
Segmented clusters of the cerebrospinal fluid system with significant extension during dehydration compared to hyperhydration in the third, fourth, and both lateral ventricles (indicated by the color code) obtained with VBM. The upper row shows the results as grayscale Maximum Intensity Projection onto the standard SPM glass brain in coronal, axial and sagittal view.

Gray matter analysis shows significant volume decrease due to dehydration in the left caudate nucleus and right-cerebellar posterior lobe, as presented in Figure 1. Figure 2 shows clusters with significant expansion of the WM during hyperhydration as compared to dehydration. Table 3 shows the cluster corrected *p*-, *t*- and *z*-values, cluster extent, and additionally the MNI coordinates of significant clusters, as shown in Figures 1 and 2. Clusters are located bilaterally in the temporal lobes and sub-gyral parietal areas, the left inferior orbito-frontal region, and the extra-nuclear region. In addition, Figure 3 shows the extent of both lateral, the third, and the fourth ventricle during dehydration as compared to hyperhydration on segmented images of CSF. All VBM results show clusters (color-coded in yellow), which remained significant after family-wise error correction (*p*<0.05) and correction for non-stationarity.

**Table 3:** Significant results of the VBM gray and white matter analysis using an 8-mm^3^ smoothing kernel and non-stationarity correction, as it is shown in Figures 1 and 2.

*p*_FWE_	*k*_e_	*t*-value	*z*-value	MNI		
				*x* [mm]	*y* [mm]	*z* [mm]
**0.005**	**638**	**6.21**	**4.53**	**−14**	**24**	**0**
**0.018**	**184**	**5.62**	**4.26**	**−2**	**−72**	**−27**
**0.025**	**378**	**5.53**	**4.22**	**6**	**−72**	**−27**
0.000	4823	7.06	4.89	−48	−39	34
0.001	1201	6.48	4.65	−8	2	1
0.014	479	6.27	4.56	−38	29	−9
0.000	1057	6.09	4.48	−40	−16	−17
0.001	765	5.68	4.29	50	−18	3
0.000	2630	5.50	4.20	20	−46	57
0.001	1010	5.22	4.06	39	−4	−32

Cluster-corrected *p*-, *t*- and *z*-values, cluster extent, *k*_e_ (in voxels) and MNI coordinates of significant clusters found in GM and WM in hyperhydration compared to dehydration. GM results are presented in bold.

**Figure 4:**
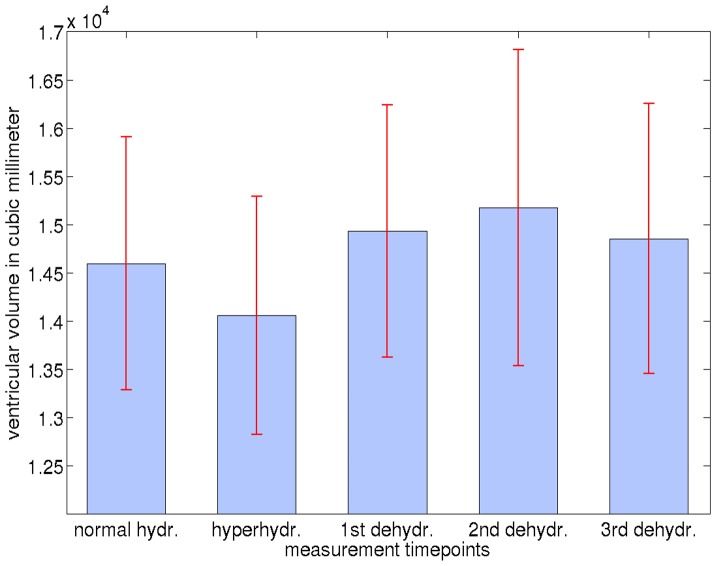
Volume of the ventricular system in mm^3^ (sum  =  lateral +3^rd^ +4^th^ ventricle) obtained with FreeSurfer for each hydration state. Red lines indicate standard error.

**Figure 5:**
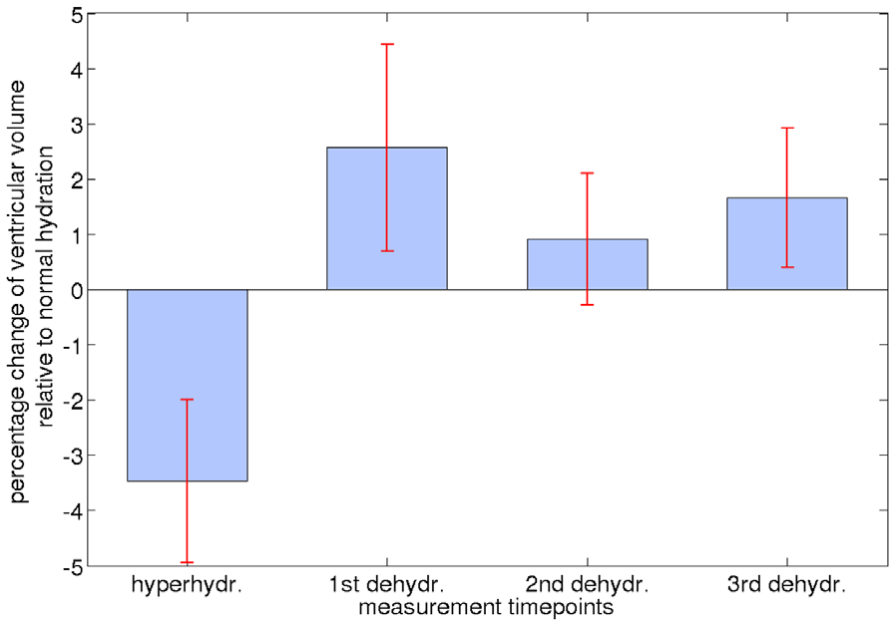
Ventricular volume change, obtained using FreeSurfer segmentation results, in percent in comparison to normal hydration (set to 100%). Red lines indicate standard error.

Figure 4 and Table 4 shows the summed volumes of the third, fourth and both lateral ventricles segmented with FreeSurfer for all hydration states. Due to technical issues, the second measurement during dehydration had to be skipped for the fifth subject. The segmentation results were therefore analyzed in two different ways using repeated measurements ANOVA. In the first approach, statistical analysis was performed without subject 5, whereas in the second approach, the ANOVA was performed without the second measurement during dehydration. The results show a statistically significant effect with both approaches: *F*(4,16) = 4.54, *p* = 0.012 and *F*(3,15) = 7.37, *p* = 0.003, respectively. Percentage increase of the ventricular volume in comparison to the baseline scan under normal hydration is shown in Figure 5.

**Table 4:** Volume of the ventricular system in ml obtained with FreeSurfer for each subject.

Subject	‘Normally hydrated’	Hyper-hydrated	Dehydrated		
	*t* = 0	*t* = 10 h	*t* = 48 h	*t* = 53 h	*t* = 58 h
1	11.797	11.492	11.915	11.568	11.682
2	18.450	17.802	19.096	18.934	19.379
3	17.228	17.185	18.114	17.963	17.856
4	10.884	10.884	11.820	11.011	11.335
5	12.571	11.995	12.852	*	12.661
6	16.645	14.998	15.800	16.388	16.208
Mean ± SD	14.6±3.2	14.1±3.0	14.9±3.2	15.2±3.7	14.9±3.4

SD is standard deviation. Missing volume data are indicated by an asterisk.

One-tailed paired *t*-tests comparing the individual measurements during dehydration against hyperhydration (set to 100%) revealed significant differences for all tests (dehydr._1_: average volume change, Δ*v* = 6.2±1.8%, *t* = 8.67, *p*<0.0002; dehydr._2_: Δ*v* = 4.4±3.6%, *t* = 2.73, *p*<0.027; dehydr._3_ Δ*v* = 5.4±2.7%, *t* = 4.83, *p*<0.004). In a real-life scenario, it is highly improbable that intentional hyperhydration is followed by unintentional dehydration. Rather, the latter, unintentional, bias is the more relevant. To assess hydration effects more realistically, we accordingly compared dehydration to “normal hydration” (first scan) status. While the ventricular volume consistently increased from hyperhydration to dehydration in the first analysis, changes between normal hydration (set to 100%) and dehydration were more subtle, showing more variation between subjects. For this comparison (i.e., when setting the baseline result to 100%), changes during dehydration did not reach significance in one-tailed paired *t*-tests (dehydr._1_: Δ*v* = 2.6±4.6%, *t* = 1.37, *p*<0.115 | dehydr._2_: Δ*v* = 0.9±2.7%, *t* = 0.77, *p*<0.243 | dehydr._3_: Δ*v* = 1.7±3.1%, *t* = 1.31, *p*<0.124). The significant average volume change during hyperhydration compared to normal hydration was –3.5±3.6% (*t = *–2.35, *p<*0.034). We note that inclusion of images with different contrast, for example *T*_2_-weighted scans, might improve the sensitivity due to an improved contrast between CSF and surrounding tissues and, hence more accurate CSF segmentation results. Analysis of dynamic changes during dehydration to investigate the effect described by Gullans and Verbalis [21] in rats did not yield additional significant effects (not shown). Furthermore, consideration of the subjects’ individual fluid intake in the statistical analysis did not improve the results.

**Figure 6:**
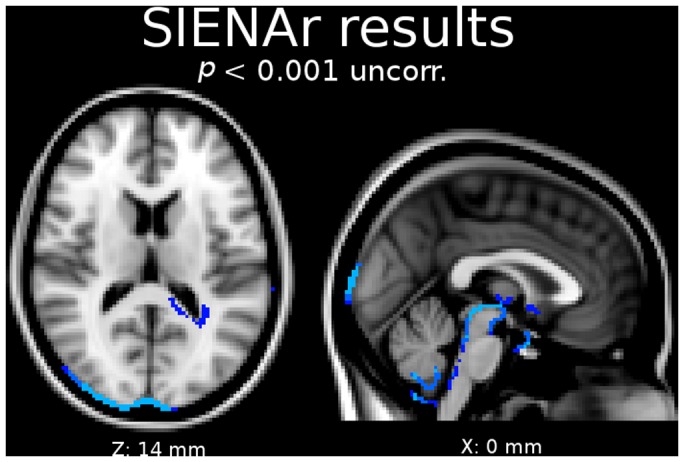
Blue color shows effects due to dehydration in regions of the third, fourth, and right lateral ventricle computed with SIENAr (voxel level, uncorrected). Additionally, effects in the region of the occipital cortex were detected.

SIENAr results did not show significant edge motions due to dehydration if statistics were corrected for multiple comparisons. When using an uncorrected voxel threshold (Figure 6), dehydration effects were observed in similar regions as described by Kempton et al. [16]. In our cohort, main effects were obtained around the 3^rd^ and 4^th^ ventricle and the brain stem, and minor effects were visible around the right lateral ventricle. Additional edge motions were detected in occipital areas.

Statistical computation of the mixed-effects model for detecting cortical thickness changes with FreeSurfer did not yield significant results for hyperhydration (assuming thickening of the cortical surface) compared to dehydration (assuming thinning of the cortical surface).

## Discussion

With the current study, we demonstrate structural brain changes due to dehydration separately for all major brain compartments. Consistent changes upon dehydration were revealed in GM, WM, and in the ventricular system employing VBM and its GLM approach. Additionally, we assessed the reliability of VBM in investigating CSF by using FreeSurfer as a reference method. SINEAr results agreed with previously published findings, when using an uncrorrected voxel threshold. FreeSurfer measurements of cortical thickness did not detect significant changes in hyperhydration compared to dehydration.

Gray matter volume reductions due to dehydration compared to hyperhydration were found using VBM in the left caudate nucleus and right-cerebellar posterior lobe. Although the left caudate nucleus is also one of the regions that are affected in AD [4], [5], we are not aware of any study suggesting a particular usage of this region in the diagnosis of AD or assessment of disease progression.

Cortical GM changes resulting in thinning or thickening due to, respectively, dehydration or hyperhydration were not detected using FreeSurfer. This is most probably due to the small sample size combined with the rather weak effect of thinning and thickening in different hydration states. Reducing the voxel size and thereby reducing partial-volume effects and improving cortex segmentation might further help to detect cortical thickness changes [27]. White matter volumetric results showed larger and more widespread results compared to GM. We found a significant decrease of tissue volume during dehydration compared to hyperhydration. Moreover, affected regions largely overlap with areas of WM loss also reported in AD studies [5], such as the temporal lobe, corpus callosum, inferior longitudinal fasciculus, inferior frontal gyrus, and sub-gyral parietal lobe. Consequently, VBM studies investigating WM changes should consider controlling for the subjects’ hydration state to avoid potential confounds by dehydration.

The observed shrinkage of WM volume during dehydration is consistent with the report of a decreased apparent diffusion coefficient during dehydration [28]. This supports the assumption of a loss of WM tissue water, which reduces the available space for water diffusion.

The observation of substantial differences in the degree of hydration-related changes between GM and WM might be due to partial volume effects [27]. In particular, in folded cortical regions, a nominal spatial resolution of 1 mm as employed in our study is insufficient for a clear distinction of GM from WM/CSF, which decreases the statistical power due to high classification variability. In contrast, segmentation stability across subjects is better in many areas of WM.

Our CSF results obtained with VBM showing an increased volume of the lateral ventricles upon dehydration are consistent with previous studies based on different methodological approaches [12], [14], [16]. Furthermore, our study extends previous results by detecting volume changes in the entire ventricular system including the fourth ventricle. This observation is consistent with the expectation that a global effect like dehydration would cause cell shrinkage and osmolality changes throughout the brain and should, hence, affect the entire ventricular system [21].

Furthermore, our SIENAr results are in line with the literature [16]. One has to keep in mind that they were only based on an uncorrected voxel-wise threshold. In this context, the limited number of subjects is even more important due to the two time-point estimation approach of SIENAr: Statistical tests with SIENAr can assess only two time points, whereas our VBM study design benefits form the repeated measurements (*n* = 3) during dehydration, which increases the degrees of freedom and thereby the statistical power. This assumption is corroborated by the observation that significance was also not reached employing VBM, when the analysis was restricted to a paired *t*-test comparing the measurement during hyperhydration to the first measurement during dehydration.

A possible explanation of the–at first sight counterintuitive–finding of ventricular expansion in dehydration is discussed by Gullans and Verbalis [21]. Dehydration is accompanied by decreased blood volume (hypovolaemia) [29]. This might contribute to reduced brain volume and an associated increase of the volume of the ventricular system [14]. Acute dehydration also increases serum osmolality (hypernatremia), which generates an osmotic gradient and therefore results in an increased diffusion of water from intracellular stores into extracellular space. This process causes cell shrinkage, in particular of astrocytes, which play an important role in water transport, and thereby leads to an expansion of the ventricular system [30].

The change in ventricular volume depending on the hydration status may now be compared with observations in diseases. Dehydration would only have a negligible effect in schizophrenia and multiple sclerosis in view of reported average ventricular increases by 26% [31] or 20–26% [32], [33], respectively. However, one year follow-up studies showed volume increases by 5–16% in AD patients and by 3.5% in MCI patients [2]. Such changes are of the same order as the increase of 6.2±1.8% between hyperhydration and dehydration observed in our study. Hence, dehydration might be misclassified as a consequence of AD or MCI.

In a recent study with different subgroups of Parkinson’s disease, ventricular enlargement ranged between 7 and 25%, depending on subgroups [34]. Although these changes exceeded those observed in our study, an influence from different fluid balances on misclassification cannot be excluded.

Finally, volume changes due to dehydration as obtained by VBM were consistent with simultaneously obtained FreeSurfer results. We may thus conclude that VBM reliably permits detection of subtle changes in the CSF system. It may thus be applied not only for GM and WM segmentation but additionally in studies of CSF.

Based on the above discussion, control of the hydration status is indicated when investigating GM and WM volume or the ventricular system. However, volume change due to hyperhydration followed by long-term dehydration is not a realistic scenario for disease studies. Involuntary dehydration starting from a normal hydration state seems more likely, as summarized in Figure 4. Thus, a ventricular expansion by approximately 2.6% after 40 hours of thirsting may be assumed to be a realistic amount of uncertainty in studies investigating the ventricular system without correcting for the hydration status. Similar results can be expected for GM and WM volume changes.

Comparable reductions of changes were described in animal studies [21] demonstrating a steady decline of the dehydration effect on the ventricular system in rats. After 21 days of thirsting, differences to baseline could no longer be observed. Such a regulation might be the same for the whole human brain and therefore long-lasting dehydration might result in a smaller impact compared to the maximum increase from hyperhydration to dehydration revealed in this study. A simple possibility to correct for the acute form of the dehydration confound is to ensure a certain amount of fluid intake of every subject in advance of scanning. Instructions describing guidelines for sufficient fluid intake approximately 24 hours before scanning should diminish dehydration effects and establish improved comparability between subjects [21].

## References

[1] Kinkingnehun S, Sarazin M, Lehericy S, Guichart-Gomez E, Hergueta T, et al (2008) VBM anticipates the rate of progression of Alzheimer disease: a 3-year longitudinal study. Neurology 70: 2201–2211. 18448872 10.1212/01.wnl.0000303960.01039.43

[2] Nestor SM, Rupsingh R, Borrie M, Smith M, Accomazzi V, et al (2008) Ventricular enlargement as a possible measure of Alzheimer's disease progression validated using the Alzheimer's disease neuroimaging initiative database. Brain 131: 2443–2454. 18669512 10.1093/brain/awn146 PMC2724905

[3] Bendfeldt K, Kuster P, Traud S, Egger H, Winklhofer S, et al (2009) Association of regional gray matter volume loss and progression of white matter lesions in multiple sclerosis - A longitudinal voxel-based morphometry study. Neuroimage 45: 60–67. 19013533 10.1016/j.neuroimage.2008.10.006

[4] Karas GB, Scheltens P, Rombouts SA, Visser PJ, van Schijndel RA, et al (2004) Global and local gray matter loss in mild cognitive impairment and Alzheimer's disease. Neuroimage 23: 708–716. 15488420 10.1016/j.neuroimage.2004.07.006

[5] Guo X, Wang Z, Li K, Li Z, Qi Z, et al (2010) Voxel-based assessment of gray and white matter volumes in Alzheimer's disease. Neuroscience Letters 468: 146–150. 19879920 10.1016/j.neulet.2009.10.086 PMC2844895

[6] Schott JM, Price SL, Frost C, Whitwell JL, Rossor MN, et al (2005) Measuring atrophy in Alzheimer disease: a serial MRI study over 6 and 12 months. Neurology 65: 119–124. 16009896 10.1212/01.wnl.0000167542.89697.0f

[7] Schroeter ML, Stein T, Maslowski N, Neumann J (2009) Neural correlates of Alzheimer's disease and mild cognitive impairment: a systematic and quantitative meta-analysis involving 1351 patients. Neuroimage 47: 1196–1206. 19463961 10.1016/j.neuroimage.2009.05.037 PMC2730171

[8] Frisoni GB, Fox NC, Jack CR, Scheltens P, Thompson PM (2010) The clinical use of structural MRI in Alzheimer disease. Nature Reviews Neurology 6: 67–77. 20139996 10.1038/nrneurol.2009.215 PMC2938772

[9] Bendel P, Koivisto T, Aikia M, Niskanen E, Kononen M, et al (2010) Atrophic enlargement of CSF volume after subarachnoid hemorrhage: correlation with neuropsychological outcome. AJNR Am J Neuroradiol 31: 370–376. 19942696 10.3174/ajnr.A1804 PMC7964141

[10] Koo MS, Dickey CC, Park HJ, Kubicki M, Ji NY, et al (2006) Smaller neocortical gray matter and larger sulcal cerebrospinal fluid volumes in neuroleptic-naive women with schizotypal personality disorder. Arch Gen Psychiatry 63: 1090–1100. 17015811 10.1001/archpsyc.63.10.1090

[11] Hagemann G, Ugur T, Schleussner E, Mentzel HJ, Fitzek C, et al (2011) Changes in brain size during the menstrual cycle. PLoS One 6: e14655. 21326603 10.1371/journal.pone.0014655 PMC3033889

[12] Duning T, Kloska S, Steinstrater O, Kugel H, Heindel W, et al (2005) Dehydration confounds the assessment of brain atrophy. Neurology 64: 548–550. 15699394 10.1212/01.WNL.0000150542.16969.CC

[13] Schols JM, De Groot CP, van der Cammen TJ, Olde Rikkert MG (2009) Preventing and treating dehydration in the elderly during periods of illness and warm weather. J Nutr Health Aging 13: 150–157. 19214345 10.1007/s12603-009-0023-z PMC12877542

[14] Dickson JM, Weavers HM, Mitchell N, Winter EM, Wilkinson ID, et al (2005) The effects of dehydration on brain volume – preliminary results. Int J Sports Med 26: 481–485. 16037892 10.1055/s-2004-821318

[15] Kempton MJ, Ettinger U, Foster R, Williams SC, Calvert GA, et al (2011) Dehydration affects brain structure and function in healthy adolescents. Human Brain Mapping 32: 71–79. 20336685 10.1002/hbm.20999 PMC6869970

[16] Kempton MJ, Ettinger U, Schmechtig A, Winter EM, Smith L, et al (2009) Effects of acute dehydration on brain morphology in healthy humans. Human Brain Mapping 30: 291–298. 18064587 10.1002/hbm.20500 PMC6871128

[17] Smith SM, Zhang Y, Jenkinson M, Chen J, Matthews PM, et al (2002) Accurate, robust, and automated longitudinal and cross-sectional brain change analysis. Neuroimage 17: 479–489. 12482100 10.1006/nimg.2002.1040

[18] Barta PE, Dhingra L, Royall R, Schwartz E (1997) Improving stereological estimates for the volume of structures identified in three-dimensional arrays of spatial data. J Neurosci Methods 75: 111–118. 9288642 10.1016/s0165-0270(97)00049-6

[19] Ashburner J, Friston KJ (2000) Voxel-based morphometry–the methods. Neuroimage 11: 805–821. 10860804 10.1006/nimg.2000.0582

[20] Armstrong LE, Pumerantz AC, Fiala KA, Roti MW, Kavouras SA, et al (2010) Human hydration indices: acute and longitudinal reference values. Int J Sport Nutr Exerc Metab 20: 145–153. 20479488 10.1123/ijsnem.20.2.145

[21] Gullans SR, Verbalis JG (1993) Control of brain volume during hyperosmolar and hypoosmolar conditions. Annu Rev Med 44: 289–301. 8476251 10.1146/annurev.me.44.020193.001445

[22] Worsley KJ, Andermann M, Koulis T, MacDonald D, Evans AC (1999) Detecting changes in nonisotropic images. Human Brain Mapping 8: 98–101. 10524599 10.1002/(SICI)1097-0193(1999)8:2/3<98::AID-HBM5>3.0.CO;2-F PMC6873343

[23] Hayasaka S, Phan KL, Liberzon I, Worsley KJ, Nichols TE (2004) Nonstationary cluster-size inference with random field and permutation methods. Neuroimage 22: 676–687. 15193596 10.1016/j.neuroimage.2004.01.041

[24] Hayasaka S, Nichols TE (2003) Validating cluster size inference: random field and permutation methods. Neuroimage 20: 2343–2356. 14683734 10.1016/j.neuroimage.2003.08.003

[25] Fischl B, Salat DH, Busa E, Albert M, Dieterich M, et al (2002) Whole brain segmentation: automated labeling of neuroanatomical structures in the human brain. Neuron 33: 341–355. 11832223 10.1016/s0896-6273(02)00569-x

[26] Worsley KJ, Taylor JE, Carbonell F, Chung MK, Duerden E, et al.. (2009) SurfStat: A Matlab toolbox for the statistical analysis of univariate and multivariate surface and volumetric data using linear mixed effects models and random field theory. OHBM poster.

[27] Tardif CL, Collins DL, Pike GB (2010) Regional impact of field strength on voxel-based morphometry results. Human Brain Mapping 31: 943–957. 19862698 10.1002/hbm.20908 PMC6871228

[28] Righini NC, Narring F, Navarro C, Perret-Catipovic M, Ladame F, et al (2005) Antecedents, psychiatric characteristics and follow-up of adolescents hospitalized for suicide attempt of overwhelming suicidal ideation. Swiss Med Wkly 135: 440–447. 16208581 10.4414/smw.2005.10754

[29] Berk L, Rana S (2006) Hypovolemia and dehydration in the oncology patient. J Support Oncol 4: 447–454; discussion 455–447. 17080733

[30] Simard M, Nedergaard M (2004) The neurobiology of glia in the context of water and ion homeostasis. Neuroscience 129: 877–896. 15561405 10.1016/j.neuroscience.2004.09.053

[31] Wright IC, Rabe-Hesketh S, Woodruff PW, David AS, Murray RM, et al (2000) Meta-analysis of regional brain volumes in schizophrenia. Am J Psychiatry 157: 16–25. 10618008 10.1176/ajp.157.1.16

[32] Brex PA, Jenkins R, Fox NC, Crum WR, O'Riordan JI, et al (2000) Detection of ventricular enlargement in patients at the earliest clinical stage of MS. Neurology 54: 1689–1691. 10762518 10.1212/wnl.54.8.1689

[33] Turner B, Ramli N, Blumhardt LD, Jaspan T (2001) Ventricular enlargement in multiple sclerosis: a comparison of three-dimensional and linear MRI estimates. Neuroradiology 43: 608–614. 11548165 10.1007/s002340000457

[34] Apostolova LG, Beyer M, Green AE, Hwang KS, Morra JH, et al (2010) Hippocampal, caudate, and ventricular changes in Parkinson's disease with and without dementia. Mov Disord 25: 687–695. 20437538 10.1002/mds.22799 PMC3068920

